# Biejiajian Pill Inhibits Carcinogenesis and Metastasis via the Akt/GSK-3β/Snail Signaling Pathway in Hepatocellular Carcinoma

**DOI:** 10.3389/fphar.2021.610158

**Published:** 2021-03-08

**Authors:** Jialing Sun, Weicong Chen, Bin Wen, Mingjia Zhang, Haitao Sun, Xuemei Yang, Wenting Zhao, Lei La, Haiyan An, Jie Pang, Lei Gao, Songqi He

**Affiliations:** ^1^ School of Traditional Chinese Medicine, Southern Medical University, Guangzhou, China; ^2^ Department of Traditional Chinese Medicine, The Air Force Hospital of Southern Theater Command, Guangzhou, China; ^3^ Department of Pharmacy, Nanfang Hospital, Southern Medical University, Guangzhou, China

**Keywords:** hepatocellular carcinoma, biejiajian pill, traditional Chinese medicine, akt/GSK-3β/Snail signaling pathway, epithelial-mesenchymal transition

## Abstract

Hepatocellular carcinoma (HCC) is among the most usual cancers globally. In China, Biejiajian pill (BJJP), Traditional Chinese Medicine clinical prescription, is broadly utilized for the prevention and therapy of HCC. However, the mechanisms by which BJJP exerts its effects on the prevention of tumor invasion and metastasis are still largely unknown. In this study, *in vitro* multiple hepatic cancer cell lines and an *in vivo* xenograft mice model were used to validate the preventive effects and molecular mechanisms of BJJP in HCC. We established that BJJP significantly repressed the proliferation, metastasis and infiltration of HCC cells. Furthermore, BJJP remarkably suppressed HCC cell migration, as well as invasion via epithelial-mesenchymal transition (EMT) by modulating Snail expression, which was associated with the repression of Akt/GSK-3β/Snail signaling axis activation. *In vivo* HCC xenograft results indicated that BJJP delayed HCC development and efficiently inhibited lung metastasis. Taken together, BJJP was shown to be an effective therapeutic agent against HCC through repression of the Akt/GSK-3β/Snail signaling cascade and EMT.

## Introduction

Liver cancer is the 6th most often diagnosed cancer, as well as the 3rd principal cause of cancer-linked fatalities in the world. It accounted for an estimated 0.84 million new cases, as well as 0.78 million mortalities in 2018 (Bray et al., 2018). In China, liver cancer incidence rates escalated from 21.56 to 36.52 per 100,000, whereas mortalities escalated by 44.46% (from 20.49 to 29.6) from 1990 to 2017 (Wang F. et al., 2019). In china, liver cancer incidence was ranked 4th among all cancers and was the 2nd principal cause of cancer-linked mortalities. Hepatocellular carcinoma (HCC) is expected to account for 70–85% of liver cancers around the world. HCC shows poor prognosis, with a low 5-years relative survival rate of about 12.1% in China (Zheng et al., 2018). Therefore, the identification of effective ways to prevent HCC and reduce its mortality has become an urgent need worldwide.

Although many significant advancements have been achieved for the clinical treatment of HCC, the morbidity and HCC mortality rate remain high. Due to inconspicuous symptoms during the initial period, most patients are diagnosed at the advanced stage, by which the possibility of local treatment methods, such as therapeutic hepatectomy, tumor ablation or tranhepatic arterial intervention, is not available. In terms of treatment, although HCC can be effectively treated using surgical resection and liver transplantation, only 5–15% of patients are fit for surgical resection, as it is only appropriate for early stage patients (Doycheva and Thuluvath, 2019; Anwanwan et al., 2020). The prognosis of HCC has remained poor, primarily because of high levels of tumor relapse and distant metastasis after various treatment methods (Jiang et al., 2018). Therefore, it is necessary for us to explore new strategies for the therapy of HCC.

Epithelial–mesenchymal transition (EMT) is referred to as the process in which epithelial cells lose their epithelial phenotypic characteristics and gain the phenotypic features of mesenchymal cells. This is manifested by the loss of cell polarity, decreased intercellular adherence and enhanced motor ability. EMT is imperative for the progression of local infiltration and distant metastasis of many tumor cells (Zhang K. et al., 2018). Previous researches have demonstrated that the Akt/GSK-3β/Snail signaling axis participates in the metastasis of various types of tumors via modulating EMT (Liu et al., 2014). In this pathway, the activation of Akt increases the phosphorylation of the 9^th^ residue of GSK-3β, which facilitates GSK-3β ubiquitination and suppresses the degradation of Snail, stimulating Snail protein stabilization, as well as nuclear localization, ultimately promoting EMT (Lan et al., 2018).

Traditional Chinese Medicine (TCM) can serve an essential role for the prevention and treatment of HCC. Studies have shown that TCM can relieve clinical manifestations, improve quality of life, enhance immune function, intercept relapse, as well as metastasis, obstruct cancer progression, and extend survival (Guo et al., 2015). TCM considers HCC as Jiju, Zhengjia, with a complex pathogenesis involving multiple systems, tissues and organs, cold-heat mixing, and intermingled deficiency and excess. In view of the pathogenic characteristics of HCC, prevention and treatment principles of TCM involve three aspects, namely, Fuzheng peiben, resolve toxins and dissipate bonds and promote blood circulation to remove blood stasis (Wang et al., 2015). Moreover, an increasing number of studies have used TCM as targeted treatments for EMT-induced cancer progression during recent years (Liu et al., 2018).

Biejiajian pill (BJJP), a renowned and canonical Chinese medicinal formula from “The Synopsis of Golden Chamber,” consists of 23 ingredients (turtle shell, donkey-hide gelatin, nidus vespae, pillbug, ground beetle, dung beetle, saltpeter, bupleurum, scutellaria, pinellia, codonopsis, *rhizoma zingiberis*, *magnolia officinalis*, cassia twig, radix paeoniae alba, rhizoma belamcandae, peach kernel, cortex moutan, *rheum officinale*, trumpet creeper, semen lepidii, pyrrosia leaf, fringed pink). According to TCM, BJJP can promote circulation, relieve stasis, improve immunity, eliminate pathogenic factors, as well as disperse and soften hard masses; therefore, it is suitable for patients with liver fibrosis and cirrhosis (Yao et al., 2004; Li M. et al., 2018). Some of the ingredients, such as rhubarb (Wu et al., 2017) and radix bupleuri (Wang et al., 2018), have been shown to inhibit tumor proliferation and metastasis via interruption of cellular signaling pathways, such as PI3K/Akt, MAPK/ERK, and Wnt/β-catenin. Importantly, BJJP has been shown to exert positive effects for the clinical treatment of HCC. However, the specific antitumor mechanism of BJJP is still unclear.

In this study, we first used high resolution mass spectrometry to analyze the main components of BJJP. Then, we demonstrated that BJJP could serve a pivotal function in HCC infiltration, as well as EMT of HCC cells. Additional research revealed that BJJP could inhibit the growth of subcutaneous tumors and pulmonary metastasis of orthotopic transplantation tumors *in vivo*. Both *in vitro*, as well as *in vivo* assays demonstrated that BJJP exerted a tight inhibitory influence on the invasion, as well as metastasis of HCC, and that its molecular mechanism was associated with the inhibition of EMT by BJJP via the Akt/GSK-3β/Snail cascade.

## Materials and Methods

### The Study of BJJP Using High Resolution Mass Spectrometry

The main components in each batch of BJJP were identified and analyzed using high resolution mass spectrometry. Six different batches of BJJP (lot Numbers: 180930, 181020, 180020, 191020, 191500, and 191170) were purchased from Sinopharm Zhonglian Pharmaceutical Co., Ltd. (Wuhan, China). A 1 g sample from each batch was weighed, 1 ml methanol: water (8:2, V: V) was added, and the mixture was mixed through vortex. Then the resulting mixture was ground for 6 min, then centrifuged for 10 min at 4°C using a centrifugal force of 20,000 × g. We filtered the supernatant through a 0.22 μm filter membrane, and the evaluated the filtrate. The conditions used for mass spectrometry consisted of: ion source: electrospray ionization source (ESI); scanning mode: positive, as well as negative ion switching scanning; detection approach: full mass/dd-ms^2^; resolution: 70,000 (full mass), 17,500 (dd-ms^2^); scan range: 100.0–1500.0 m/z; spary voltage: 3.8 kv (Positive); capillary temperature: 300°C; colliding gas: high purity argon (purity ≥99.999%); sheath gas: nitrogen (purity ≥99.999%), 40 Arb; aus gus heater temperature: nitrogen (purity ≥99.999%), 300°C; data acquisition time: 27.0 min. The chromatographic conditions used were as follows: chromatographic column: RP-C18 150 × 2.1 mm, 1.8 μm, Welch; flow rate: 0.300 ml/min; aqueous phase: 0.1% formic acid solution; organic phase: 0.1% formic acetonitrile; needle washing liquid: methanol; column chamber temperature: 35°C; automatic sampler temperature: 10.0°C; injection needle height: 2.00 mm; automatic sampler cleaning settings: both; washing needle volume of automatic sampler: 200.00 μl; soaking time for automatic injection needle cleansing: 3.00 ms; auto injector injection volume: 10.00 μl. The data collected under high-resolution liquid quality were initially sorted using CD2.1 (Thermo Fisher Scientific) and then retrieved and contrasted with the repository (mzCloud, mzVault, ChemSpider).

### Preparation of Serum Containing BJJP

We obtained 40 Wister rats (20 females and 20 males) weighing 250 ± 20 g each from the Experimental Animal Center of Southern Medical University (animal license no.: SCXK (yue) 2011-0015). The Wister rats were kept at room temperature (21–25°C), 50–60% relative humidity, and under 12 h cycles of day/night. The rats were provided exclusive access to water and food. Experimental operations were carried out as per the Guidelines for the Care and Use of Laboratory Animals of Southern Medical University. The rats were clustered randomly into a negative control arm (NC arm), a low-dose (L arm), a medium-dose (M arm), a high-dose (H arm), and a positive sorafenib control arm (P arm), with eight rats in each arm. BJJP was dissolved in sterilized normal saline and made into a suspension. The L, M and H arm rats were given 0.55, 1.1, and 2.2 g/kg, respectively, of concentrated BJJP through gastrogavage. The NC arm was gavaged with an equivalent amount of normal saline, while the P arm was given 0.03 g/kg sorafenib (BXHZCL3, Bayer, Germany) in normal saline. Each rat was given 2 ml of the respective solution through gastric gavage twice a day, with a gavage interval of 12 h and a total of 7 consecutive gavages. Blood samples were taken 1 h after the last gastrogavage from the abdominal aorta. The blood samples were allowed to stand for 4 h at 4°C, then centrifugation for 15 min at 3,000 r/min done, and the serum was separated under aseptic conditions. Inactivation of the separated serum done for 30 min in a 56°C water bath. After that, filtration by a 0.22 μm microporous membrane was conducted, and then kept at −80°C until used.

### Cell Lines and Cell Cultures

The MHCC-97H, as well as SMMC-7721 human HCC cell lines were bought from the Cell Bank of Chinese Academy of Sciences (Shanghai Institutes for Biological Sciences, Shanghai, China). Incubation of the MHCC-97H, as well as SMMC-7721 cells was done at 37°C using Dulbecco’s modified Eagle’s medium (DMEM, Gibco, United States, 10829018) enriched with fetal bovine serum (10%) (FBS, Gibco, United States), as well as penicillin/streptomycin (1%) (Gibco, United States, 15140122) in incubator with 5% CO_2_.

### Cell Viability Inspection

The impact of BJJP on the viability of HCC cells was inspected by a Cell Counting Kit-8 (CCK-8, GK10001, GLPBIO, China). In brief, we planted the cells into 96-well dishes with 5 × 10^4^ cells per well and cultivated for 24 h until they adhered. Incubation of the cells was done in 100 μl of DMEM containing 10% of various concentrations of BJJP or sorafenib serum for 24, 48, and 72 h. For this assay, we used two control arms: cells in the blank control arm were exposed to DMEM containing 10% FBS, while cells in the negative control arm were incubated in DMEM containing 10% rat serum. Thereafter, CCK-8 solution (10 μl) was introduced into every well, cultured for an additional 1 h in an incubator with 5% CO_2_ at 37°C. A microplate reader (Bio-tek, Winooski, Vermont, United States) was employed to establish the absorbance (OD value) of every well at the wavelength of 450 nm. Cell viability was calculated for each arm using the following formula: cell viability (%) = (OD value of treatment arm/OD value of control arm) × 100.

### Hoechst 33342/PI Fluorescent Staining

MHCC-97H and SMMC-7721 HCC cells were planted in 96-well dishes, and DMEM containing 10% of different concentrations (L, M, H) of BJJP or sorafenib serum were used for 24 h of incubation. After rinsing the cells thrice in PBS, a mixture of Hoechst33342 and PI was prepared according to instructions given in the Hoechst33342/PI Double Stain Kit (CA1120, Solaria, Beijing, China) and added for staining at 4°C for 30 min. Once again, rinsing of the cells with PBS was done, and images were captured under a fluorescence microscope. Five random regions were selected for observations and the cell apoptosis index was calculated.

### Cell Wound-Healing Assay

Plating of the MHCC-97H and SMMC-7721 HCC cells into 6-well dishes at a concentration of 2 × 10^5^ cells per well was done. Subsequently, culturing of the cells at 37°C in an incubator with 5% CO_2_. When the cells had attained 70% confluence, we removed the original medium, rinsed the cells thrice in PBS, and DMEM containing 10% of different concentrations (L, M, H) of BJJP or sorafenib serum were used for 24 h of incubation. After that, wounds were made on the confluent cell monolayer via scratching by a 20 μl pipette tip. Then, we rinsed the cells thrice using PBS, while discarding any detached cell. Thereafter, growing of the cells in DMEM was conducted. Cell migration was observed under a microscope for 0 h and 48 h in a drug serum culture. The migration area of the cells in each arm at 0 h and 48 h were analyzed using ImageJ software. The formula: cell migration area ratio = (S_0h−_S_48h_)/S_0h_, the cell migration area ratio of the HCC cells in each arm were calculated.

### Transwell Matrigel Invasion Assessment

The Transwell invasion test was conducted using 24-well transwell plates and a cell culture insert (353097, FALCON) with 8.0 μm sized pores along with a transparent PET membrane. After melting and diluting the reagent at 4°C, the Matrigel (354248, CORNING) was used to coat the filters (50 μl per filter) and 30 min incubation at 37°C conducted. The DMEM containing 10% of different concentrations (L, M, H) of BJJP or sorafenib serum were introduced to the lower chamber. After that, 5 × 10^5^ MHCC-97H, as well as SMMC-7721 cells in 1% FBS DMEM were introduced to the upper chamber, then 24 h incubation at 37°C performed. Thereafter, we used cotton swabs to completely remove cells on the upper surface of the membrane, whereas fixing of the cells that infiltrated into the lower surface was done using paraformaldehyde, and then staining using crystal violet, and the invasive cells enumerated under an inverted microscope (Nikon, Tokyo, Japan) at 200 × magnification.

### Immunofluorescence Assay

Growing of the MHCC-97H, as well as SMMC-7721 HCC cells in complete medium on chamber slides until they reached 60% confluency. Then, they were exposed to the DMEM containing 10% of different concentrations (L, M, H) of BJJP or sorafenib sera for 24 h. Rinsing of the cells was done thrice using PBS and 15 min fixing by paraformaldehyde (4%) at 4°C on glass cover slips was conducted. After that, permeabilization of the cells using Triton-X 100 (0.1%) for 10 min at 4°C was done. After being washed with PBS, blocking of the cells for 2 h using BSA at room temperature was accomplished, followed by conjugation with the primary antibodies against Phospho-Akt (Ser473; 1:200; Bioss bs-0876R), Phospho-GSK-3β (Ser9; 1:400; CST #5558) and Snail (1:200; proteintech; 13099-1-AP) via overnight-incubation at 4°C. The slides were rinsed thrice by PBS and conjugated with Alexa Fluor™ 568 goat anti-rabbit IgG (H+L) secondary antibodies (1:400; Thermo Fisher; United States; A11011) at room temperature via incubation in the dark for 1 h. Thereafter, staining of the nuclei using DAPI (Solarbio, #C0065) was performed for 10 min. Finally, we inspected the cells on a fluorescence microscope (Nikon, Tokyo, Japan).

### Reverse Transcription Quantitative PCR (RT-qPCR)

MHCC-97H and SMMC-7721 HCC cells were planted in 6-well dishes, and DMEM containing 10% of different concentrations (L, M, H) of BJJP or sorafenib serum were used for 24 h of incubation. The total RNA of these cells was then extracted using TransZol UP Plus RNA Kit (TransGen Biotech; China; ER501-01) as specification and the concentration and purity of the RNA were detected using NanoDrop™ Lite (Thermo Fisher Scientific, Waltham, MA, United States). Total RNA was reverse transcribed into cDNA by PrimeScript™ RT reagent Kit with gDNA Eraser (RR047a, Takara, Japan). According to the instruction of SYBR^®^ Premix Ex Taq™ II (Tli RNaseH Plus) (RR820a, Takara, Japan), the threshold cycle (Ct) was recorded by Light Cycler® 96 System (Roche Applied Science, Germany). 2^−△△CT^ method was used to calculated the fold changes of mRNA expression. Data were standardized to the GAPDH control and the primer of GAPDH was brought from Sangon Biotech (Shanghai) Co., Ltd. Primers of Snail, E-cadherin, N-cadherin, as well as Vimentin were synthesized by Sangon Biotech (Shanghai) Co., Ltd. and were shown in Table 1.

**TABLE 1:** Primer sequences for quantitative real-time PCR amplification.

Name	Sequence 5′-3′
Snail	F: TCG​GAA​GCC​TAA​CTA​CAG​CGA
	R: AGA​TGA​GCA​TTG​GCA​GCG​AG
E-cadherin	F: CGA​GAG​CTA​CAC​GTT​CAC​GG
	R: GGG​TGT​CGA​GGG​AAA​AAT​AGG
N-cadherin	F: TTT​GAT​GGA​GGT​CTC​CTA​ACA​CC
	R: ACG​TTT​AAC​ACG​TTG​GAA​ATG​TG
Vimentin	F: GAC​GCC​ATC​AAC​ACC​GAG​TT
	R: CTT​TGT​CGT​TGG​TTA​GCT​GGT

### Experimental Mouse Model

The mice procedures were approved by the Southern Medical University Experimental Animal Ethics Committee (Resolution No.:L2018207). The animal laboratory (Southern Medical University, Guangzhou, China) provided the four to five weeks old female BALB/c nude mice (15–16 g). These mice were given standard food and were allowed free access to tap water. For the subcutaneous tumor formation experiment, MHCC-97H cells (5 × 10^6^) were harvested in serum-free PBS. The right foreleg close to the shoulder of the mice was subcutaneous injected with 5 × 10^6^ MHCC-97H cells. After 3 days, the mice were clustered randomly into six arms (n = 6). Each arm was fed as follows: Normal Control (NC) and Model (X) arm were gavaged with normal saline; high (H), medium (M) and low (L) dose arms of BJJP were gavaged with a high dose (2.2 g/kg), medium dose (1.1 g/kg) and low dose (0.55 g/kg) of concentrated BJJP, respectively. The positive (P) control arm was administered with 0.03 g/kg sorafenib in normal saline once a day. The volume of the tumor was determined using a digital calipers following every two days. This volume was computed using the formula: volume = 0.5 × length × wide (mm^3^). The mice were sacrificed on day 28, and excision of the tumors done, followed by weighing, and imaging. For the *in vivo* lung metastasis assay, MHCC-97H HCC cells were fluorescences with lentivirus (LVCON137, Shanghai Genechem Co., Ltd.), a green fluorescent tool with GFP. Orthotopic injection of a 12.5 μl suspension [PBS and Matrigel (1:2)] containing 1 × 10^6^ LV-GFP-MHCC-97H cells into the left liver lobe of BALB/C nude mice was done. After 3 days, we randomly grouped the mice into six arms (n = 5), and were fed as follows: Normal Control (NC) and Model (X) arm were gavaged with normal saline; high (H), medium (M) and low (L) dose arms of BJJP were gavaged with a high dose (2.2 g/kg), medium dose (1.1 g/kg) and low dose (0.55 g/kg) of concentrated BJJP, respectively. The positive (P) control arm was administered with 0.03 g/kg sorafenib in normal saline once a day. Four weeks later, we sacrificed the mice, and harvested the lung and liver. Small animal bioluminescence *in vivo* imager was employed to inspect the growth, as well as lung metastasis of the liver tumors. Paraffin-embedment of the lung and liver tissues was done, and then staining using H&E for histological confirmation performed.

### Western Blotting Analysis

Lysis and homogenization of the cells or animal tissue proteins was done using the RIPA lysis buffer (P0013B, Beyotime, Shanghai, China) enriched with protease inhibitor (1%), as well as phosphatase inhibitor (1%) (all purchased from KeyGEN, Jiangsu, China) to extract total protein. After that, the protein concentrations were determined by the BCA Protein Assay Kit (P0012, Beyotime, Shanghai, China). The proteins (30 μg/lane) were fractionated on SDS-PAGE gel (12%) and transfer-embedded onto PVDF (0.45 μm membrane) (IPVH00010, Millipore, Billerica, MA, United States) membranes. Then, they were blocked using 1×TBST (0.1% Tween-20) enriched with bovine serum albumin (BSA) (5%) at room temperature (RT) for 2 h. Thereafter, overnight-incubation of the PVDF membranes at 4°C with the following primary antibodies: Akt (1:1000; CST #2920), Phospho-Akt (Ser473; 1:1000; Bioss bs-0876R), GSK-3β (1:1000; CST #12456), Phospho-GSK-3β (Ser9; 1:1000; CST #5558), E-cadherin (1:1000); Abcam ab227639), Snail (1:800; proteintech 13099-1-AP), MMP-9 (1:1000; Bimake A5725), PCNA (1:1000; Bimake A5324), Vimentin (1:1000; Servicebio GB11192), N-cadherin (1:1000; Servicebio GB11135), Cyclin D1 (1:1000; Servicebio GB13079), VEGFA (1:1000; Servicebio GB11034) and GAPDH (1:1000; Servicebio GB11002) was done. After rinsing thrice in 1 × TBST, incubation of the membranes with goat anti-rabbit IgG or goat anti-mouse IgG (all purchased from Servicebio, Wuhan, China) was done at RT for 1 h and were then again rinsed thrice with 1×TBST. Visualization of the membranes was conducted using a ECL detection kit (purchased from Millipore, Billerica, MA, United States), and the relative protein levels were measured using ImageJ software.

### Elisa Assay

The effect of BJJP on TNF-α of mice serum was detected using Elisa (CME0004, 4A Biotech, China) assay. After collecting the mouse serum, the experiment was carried out according to instructions given by the manufacturer of the assay kit.

### H&E Staining and Immunohistochemical Analysis

Fixing of the solid tumor tissues was done using paraformaldehyde (4%), followed by paraffin embedment, and chopped into 4 μm-thick slides. Deparaffination using dimethylbenzene was performed, and then the segments positioned on slides. Gradient alcohol dehydration was then conducted. Then, the staining of the sections with hematoxylin and eosin was performed for the H&E assay or antigen repaired with sodium citrate and treated according to instruction given by the manufacturer of the UltraSensitive™ S-P Immunohistochemical Staining Kit (KIT-9720, MXB, China) Thereafter, the slides were incubated with primary antibodies (*p*-Akt (1:200), *p*-GSK-3β (1:400), Snail (1:200), E-cadherin (1:100), N-cadherin (1:200), Cyclin D1 (1:200), KI67(1:1000; proteintech 27309-1-AP)) at 4°C overnight. ImageJ software was employed to statistically inspect the immunohistochemical results.

### Statistical Analysis

SPSS version 19.0 software was employed for all statistical analyses. The data were shown as mean ± SD. One-way analysis of variance (ANOVA) and two-way ANOVA were used to determine differences between arms. *p* < 0.05 denoted statistical significance.

## Results

### Finger-Print of BJJP

**FIGURE 1:**
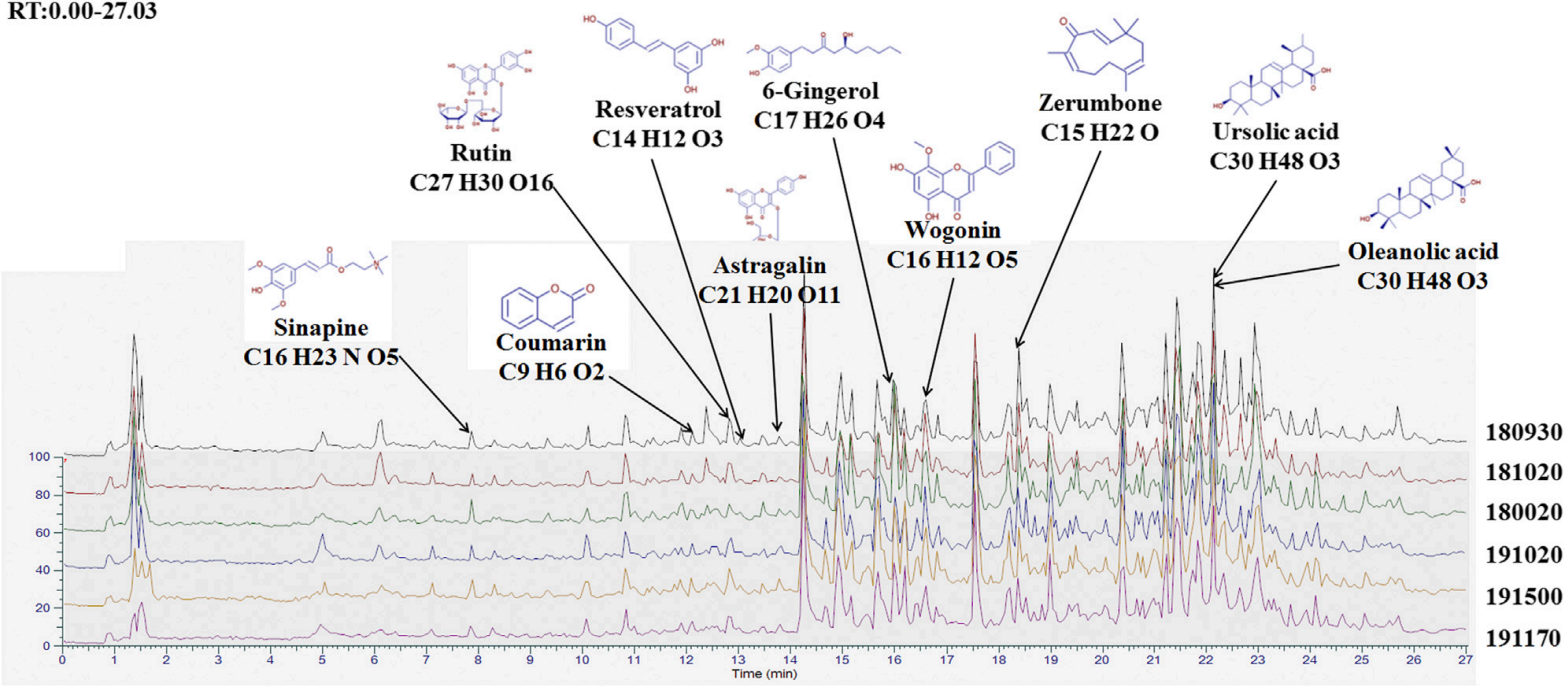
Finger-print of six different batches of Biejiajian pill (BJJP). The Finger-prints of six different batches of BJJP were determined using high resolution mass spectrometry. Ten pharmaceutical ingredients were labeled and used to analyze the structures of mass spectrometry based on chromatographic retention time.

We used high resolution mass spectrometry to analyze the main components of BJJP, to elucidate the mechanism of action of BJJP against HCC. For data obtained through mass spectrometry, we used the mzCloud, mzVault and ChemSpider databases for the retrieval and comparative analysis, and a total of 2,727 compounds were found to match. Among them, 10 pharmaceutical ingredients, Wogonin, Ursolic acid, Zerumbone, 6-Gingerol, Coumarin, Astragalin, Resveratrol, Rutin, Sinapine and Oleanolic acid, received a mzCloud Best Match score greater than 94. As shown in Figure 1, we obtained the finger-prints of 6 different batches of BJJP, and the 10 matching pharmaceutical ingredients were labeled and used to analyze the structures of mass spectrometry based on chromatographic retention time.

### BJJP Represses Proliferation and Induces Apoptosis of HCC *In Vitro*

**FIGURE 2:**
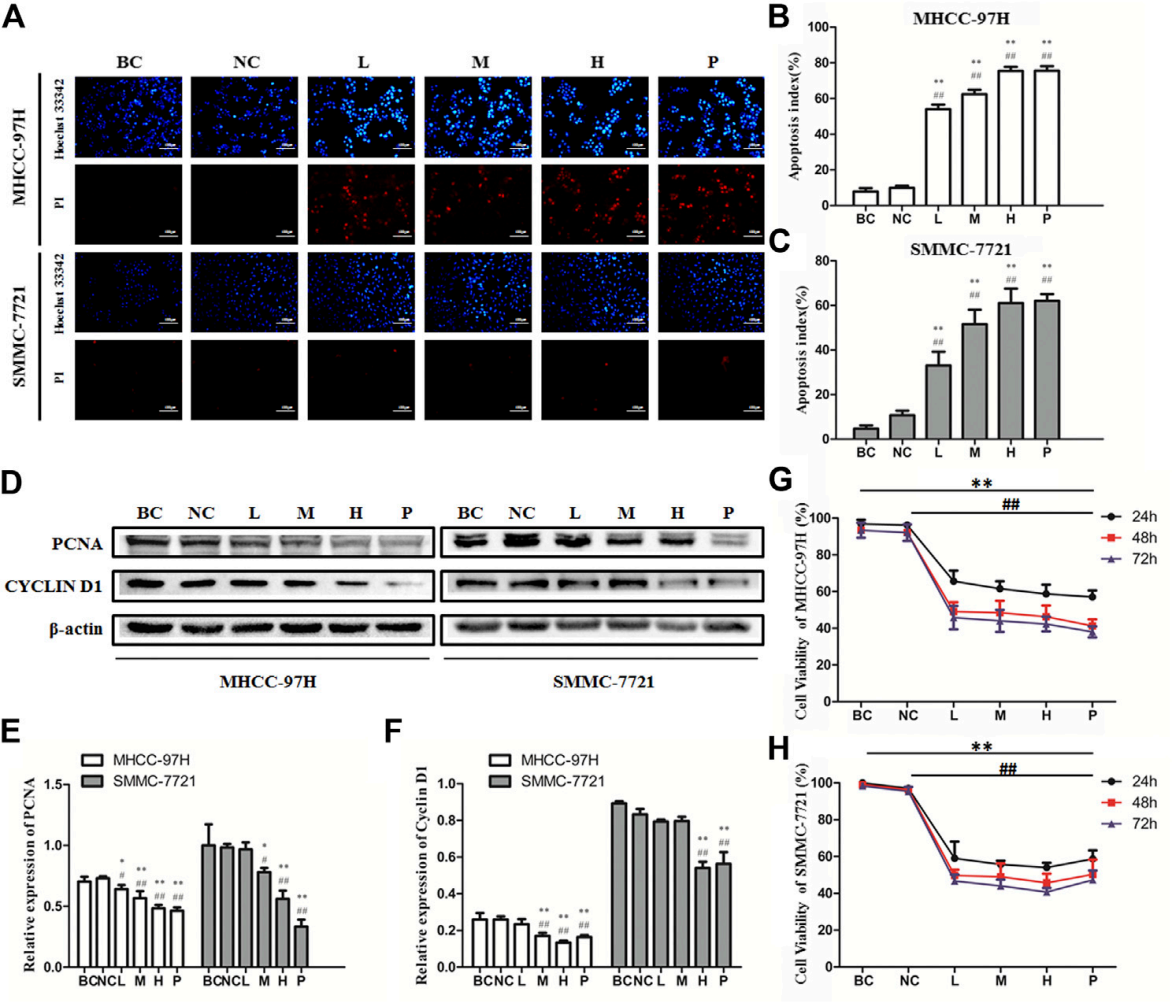
Biejiajian pill (BJJP) markedly inhibited the proliferation and induced apoptosis of MHCC-97H and SMMC-7721 cells. Cells were randomly grouped into six arms: BC control arm (cells were incubated in DMEM containing 10% FBS), NC control arm (cells were incubated in DMEM containing 10% normal rat serum), BJJP-low dose arm (L), BJJP-medium dose arm (M), BJJP-high dose arm (H), and sorafenib positive control arm (P). **(A)**: Hoechst 33342/PI fluorescent staining. Red (PI) and bright blue regions (Hoechst 33342) indicate necrotic cells and apoptosis cells, respectively **(B,C)**: Percentage of apoptosis in BJJP treated MHCC-97H and SMMC-7721 cells. **(D–F)**: After incubation with different titers of BJJP for 24 h, total proteins from the MHCC-97H and SMMC-7721 cells were inspected using western blotting analysis with antibodies specific to PCNA and CYCLIN D1. Protein expression data were standardized to the GAPDH control and are shown as mean ± SD (n = 3). **(G,H)**: The cell viability of the MHCC-97H and SMMC-7721 cells treated with different concentrations of BJJP for 24, 48, and 72 h was inspected using CCK-8 assay. Data are shown as mean ± SD (n = 3). *, *p* < 0.05; **, *p* < 0.01 compared with the BC control arm (cells were incubated in DMEM enriched with 10% FBS); #, *p* < 0.05; ##, *p* < 0.01 relative to the NC control arm (cells were incubated in DMEM containing 10% normal rat serum). One-way ANOVA was used to determine statistical significance.

The Hoechst33342/PI fluorescent staining test was employed to inspect if BJJP triggered apoptosis of the HCC cells. As illustrated in Figures 2A–C, after incubation for 24 h, BJJP dose-dependently triggered the apoptosis of both MHCC-97H and SMMC-7721 cells. The proliferative ability of HCC (MHCC-97H, as well as SMMC-7721 cells) was inspected using CCK8 assay. In brief, incubation of MHCC-97H (Figure 2G), as well as SMMC-7721 (Figure 2H) cells with DMEM containing 10% BJJP or Sorafenib for 24, 48, and 72 h decreased the cell viability in a dose-dependent, as well as time-dependent approach. In addition, we moreover inspected the expression of proliferation-linked proteins, entailing Cyclin D1, as well as PCNA, to validate the inhibitory effect of BJJP. CyclinD1 constitutes a modulatory protein of the cell cycle and serves an essential function in transition from G1 to S phase in a variety of tumor cells (Xi et al., 2019). PCNA is a DNA clamp essential for the replication of DNA and also participates in DNA excision repair and the control of the cell cycle (Kang et al., 2019). As shown in Figures 2D–F, BJJP remarkably repressed Cyclin D1, as well as PCNA expressions in a dose-dependent approach (*p* < 0.05). These data opined that BJJP suppresses the proliferation and induced the apoptosis of HCC cells.

### BJJP Inhibits Expression of VEGFA and MMP-9 in HCC Cells

**FIGURE 3:**
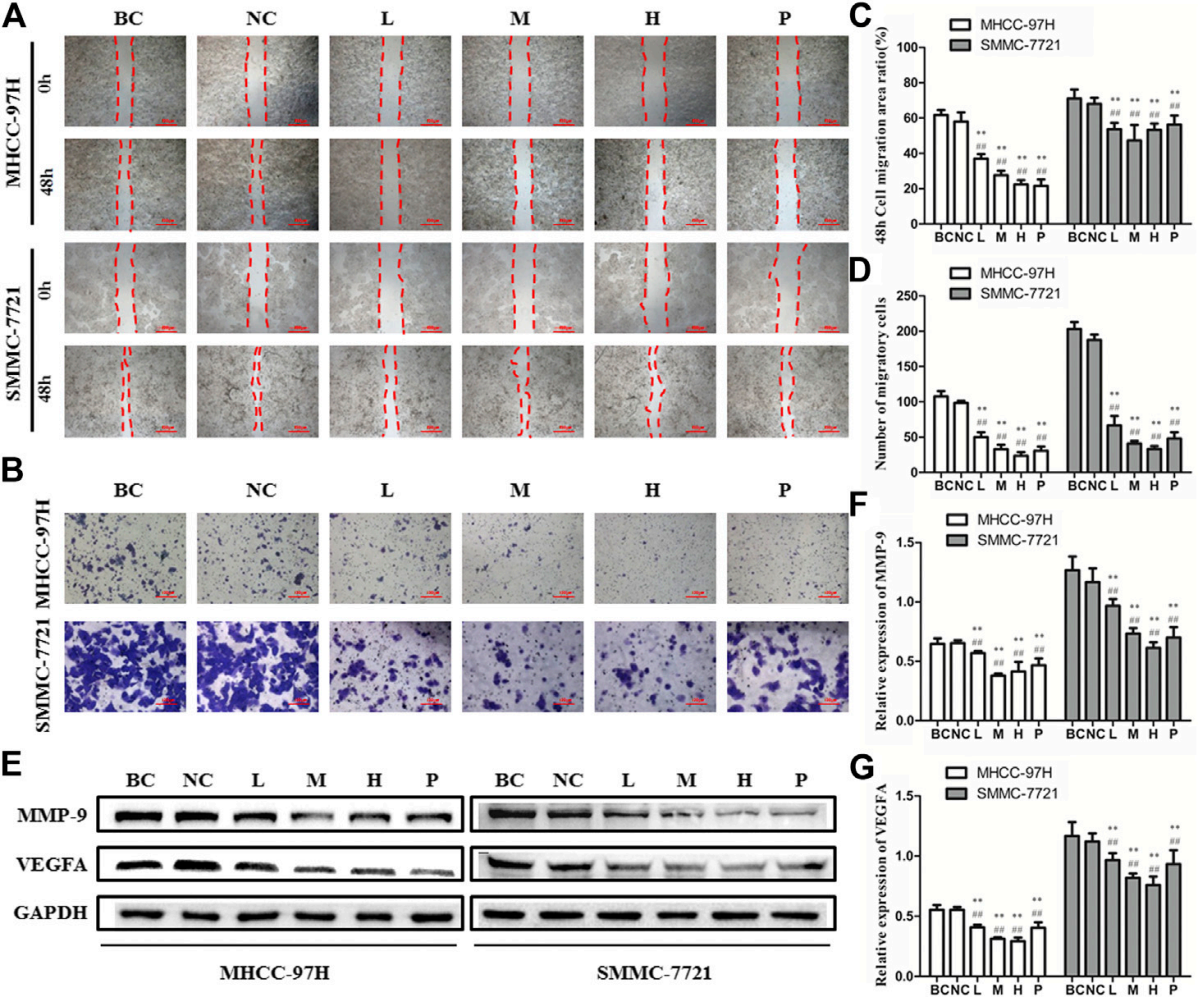
Biejiajian pill (BJJP) markedly inhibited the invasion and metastasis in MHCC-97H and SMMC-7721 cells. Cells were randomly grouped into six arms: BC control arm (cells were incubated in DMEM containing 10% FBS), NC control arm (cells were incubated in DMEM containing 10% normal rat serum), BJJP-low dose arm (L), BJJP-medium dose arm (M), BJJP-high dose arm (H), and sorafenib positive control arm (P). **(A,B)**: The influences of BJJP on the migration, as well as invasion of MHCC-97H and SMMC-7721 cells were examined using wound healing assay (scale bar, 500 μm, 40×) and Transwell assay (scale bar, 100 μm, 200×). **(C)**: 48 h cell migration area ratio (%) in BJJP treated MHCC-97H and SMMC-7721 cells. **(D)**: Number of migratory cells in BJJP treated MHCC-97H, as well as SMMC-7721 cells **(E–G)**: After incubation with different concentrations of BJJP for 24 h, total proteins from the MHCC-97H and SMMC-7721 cells were inspected using western blotting with antibodies specific to MMP-9 and VEGFA. Protein expression data were standardized to the GAPDH control. Data are shown as mean ± SD (n = 3). *, *p* < 0.05; **, *p* < 0.01 relative to the BC control arm (cells were incubated in DMEM containing 10% FBS); #, *p* < 0.05; ##, *p* < 0.01 compared with the NC control arm (cells were incubated in DMEM enriched 10% normal rat serum). One-way ANOVA was used to determine statistical significance.

To analyze the influence of BJJP on HCC cell migration, as well as invasion, we performed the wound healing, as well as Transwell migration assays using MHCC-97H and SMMC-7721 cells. As illustrated in Figures 3A–D, we found that the migration area of HCC cells and the number of invading cells passing through the membrane decreased significantly following BJJP treatment, in a dose-dependent, as well as time-dependent approach (*p* < 0.05). Since MMP-9 and VEGFA have been implicated in the progression and metastasis of various cancers, western blot assessment was employed to measure the expression of MMP-9 and VEGFA in HCC cells treated with BJJP. As shown in Figures 3E–G, BJJP significantly decreased the expression of MMP-9 and VEGFA *in vitro* (*p* < 0.05). These data implied that BJJP inhibited the invasion and metastasis of HCC cells.

### BJJP Inhibits EMT by Suppressing Initiation of the Akt/GSK-3β/Snail Axis in HCC

**FIGURE 4:**
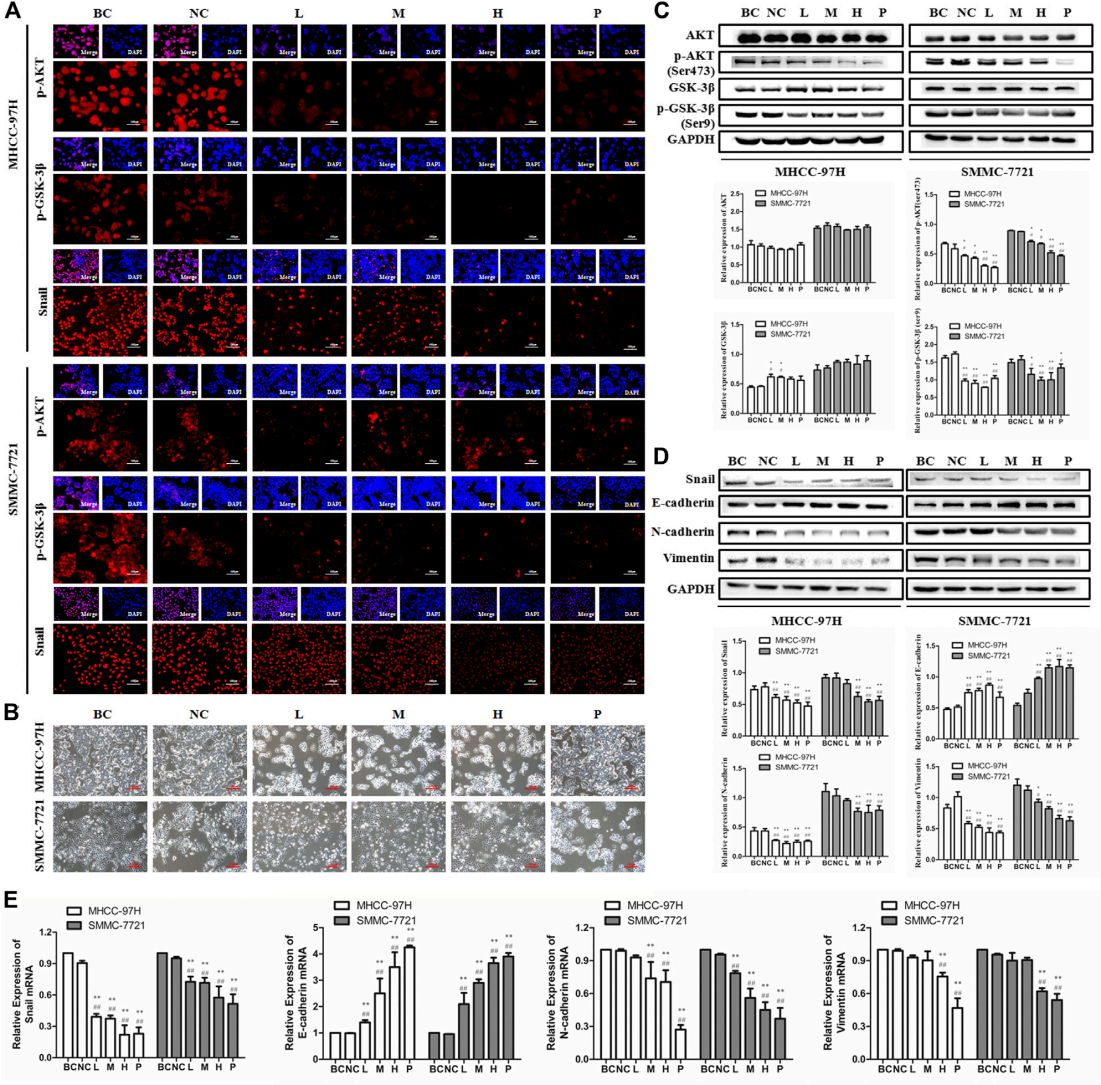
Biejiajian pill (BJJP) inhibited EMT by suppressing activation of the Akt/GSK-3β/Snail pathway in MHCC-97H and SMMC-7721 cells. Cells were randomly grouped into six arms: BC control arm (cells were incubated in DMEM containing 10% FBS), NC control arm (cells were incubated in DMEM containing 10% normal rat serum), BJJP-low dose arm (L), BJJP-medium dose arm (M), BJJP-high dose arm (H), and sorafenib positive control arm (P). **(A)**: The subcellular localization of *p*-Akt (Ser 473), *p*-GSK-3β (Ser 9) and Snail proteins in MHCC-97H, as well as SMMC-7721 cells were inspected using immunofluorescence (IF) (scale bar, 100 μm, 200×). DAPI was employed to stain the cell nuclei. **(B)** Morphological changes of MHCC-97H and SMMC-7721 cells incubated by DMEM containing 10% of different concentrations (L, M, H) of BJJP or sorafenib serum for 24 h (scale bar, 100 μm, 200×). **(C):** The expressions of Akt, *p*-Akt (Ser 473), GSK-3β and *p*-GSK-3β (Ser 9) were examined using western blotting analysis. **(D):** The expressions of E-cadherin, N-cadherin, Vimentin and Snail were examined using western blotting analysis. GAPDH was used as an internal control. Illustrative western blots and quantitative analysis of protein expression using ImageJ software are indicated **(E):** Relative mRNA levels of Snail, E-cadherin, N-cadherin and Vimentin normalized with GAPDH. Data are shown as mean ± SD (n = 3). *, *p* < 0.05; **, *p* < 0.01 compared with the BC control arm; #, *p* < 0.05; ##, *p* < 0.01 relative to the NC control arm. One-way ANOVA was used to determine statistical significance.

In EMT, epithelial cells gain the phenotypic features of mesenchymal cells and show decreased intercellular adhesion and increased motility (Zhou et al., 2004). To detect the role of BJJP in morphological changes of HCC cells, the MHCC-97H, as well as SMMC-7721 human HCC cells were incubated in DMEM containing 10% of various concentrations of BJJP or sorafenib serum for 24 h. As shown in Figure 4B, BJJP gradually change MHCC-97H cells from polygon to cobblestone shape, as well as SMMC-7721 cells from spindle shape to pebble shape. These results indicated that BJJP could inhibit mesenchymal cell phenotypic changes in HCC cells morphologically. Akt initiation serves a pivotal role in inducing EMT by promoting the inactivation of GSK-3β, which subsequently leads to Snail stabilization. Moreover, research has shown that the Akt signaling cascade is inherently active in HCC (Lan et al., 2018). Therefore, we speculated that BJJP may inhibit HCC EMT via the Akt/GSK-3β/Snail axis. As shown in Figures 4A,C,D, western blotting assays and Immunofluorescence assays demonstrated a reduction in the expression of *p*-Akt (Ser473), *p*-GSK-3β (Ser9) as well as Snail in both MHCC-97H, as well as SMMC-7721 cells treated with BJJP (*p* < 0.05). Moreover, the Immunofluorescence assays result implicated that BJJP deceased the nuclear translocation of Snail by regulating GSK-3β.These data implied that BJJP might have inhibited the initiation of the Akt/GSK-3β/Snail cascade. In addition, we further inspected the expression of EMT linked target genes in HCC cells subjected to BJJP. As Snail is a transcriptional repressor of E-cadherin (Zhou et al., 2004), we further detected the mRNA expression of Snail, E-cadherin, N-cadherin, as well as Vimentin. As illustrated in Figures 4D,E western blotting analysis and RT-PCR indicated that BJJP reduced the expression of mesenchymal biosignatures containing N-cadherin, Snail and Vimentin (*p* < 0.05), while it escalated the expression of E-cadherin, an epithelial biosignature (*p* < 0.05), validating the EMT inhibition effects of BJJP in HCC cells. All these findings further confirmed the mechanism by which BJJP moderates HCC proliferation, metastasis and invasion via the Akt/GSK-3β/Snail pathway.

### BJJP Suppresses Growth, as Well as Metastasis of HCC *In Vivo*

**FIGURE 5:**
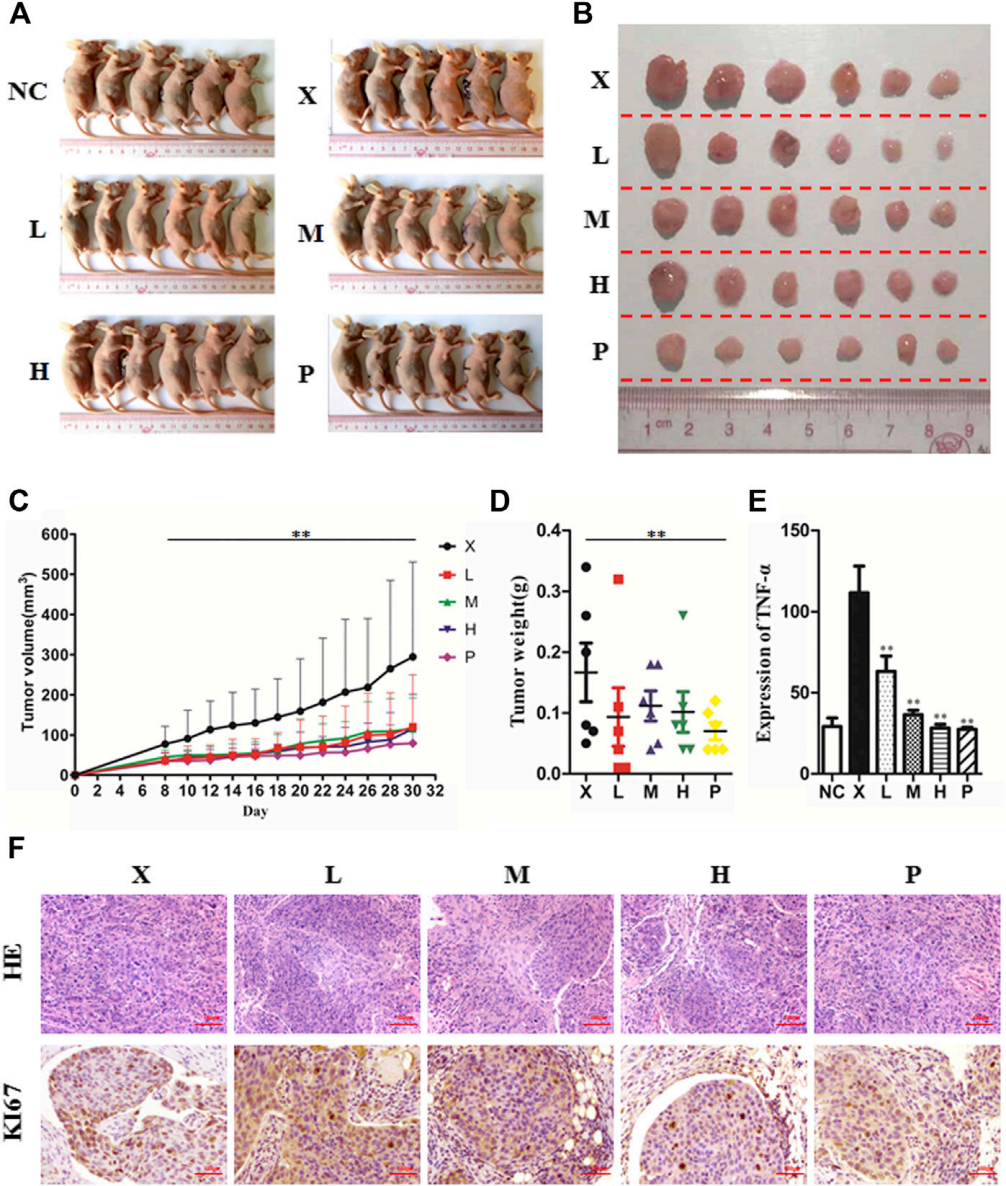
Biejiajian pill (BJJP) inhibited tumor growth of hepatocellular carcinoma *in vivo*. BALB/c nude mice were subcutaneously injected with MHCC-97H cells and subsequently treated with BJJP. Mice were randomly grouped into six arms (n = 6): Normal Control arm (NC), Model arm (X), BJJP-low dose arm (L), BJJP-medium dose arm (M), BJJP-high dose arm (H), and sorafenib positive control arm (P) **(A,B)**: Subcutaneous tumors were dissected from the nude mice and photographed (n = 6). **(C):** Tumor volumes were measured every 2 days and are presented as mean ± SD. **(D):** Tumors were weighted immediately after dissection from the nude mice. **(E):** Elisa assay indicated that BJJP significantly decreased the expression of TNF-α in mice serum. **(F):** H&E and immunohistochemical staining of the tumor tissues showed that BJJP decreased the number of KI67-positive cells in mice tumor tissues. Data are shown as mean ± SD (n = 3). *, *p* < 0.05; **, *p* < 0.01 relative to the X arm (tumor model arm). One-way ANOVA was used to determine statistical significance.

**FIGURE 6:**
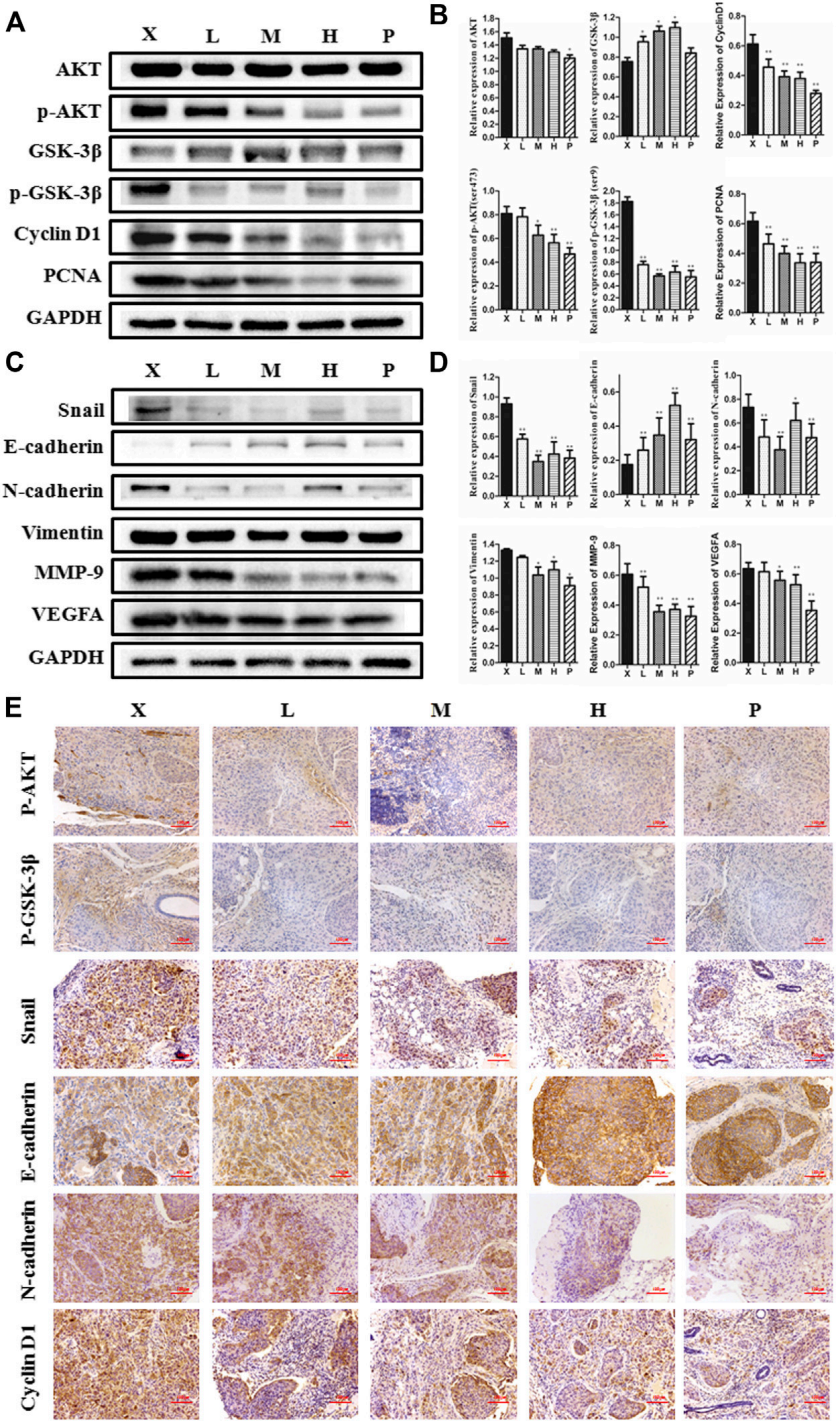
Biejiajian pill (BJJP) inhibited the growth and EMT of HCC by suppressing activation of the Akt/GSK-3β/Snail pathway *in vivo*. Mice were randomly grouped into six arms (n = 6): Normal Control arm (NC), Model arm (X), BJJP-low dose arm (L), BJJP-medium dose arm (M), BJJP-high dose arm (H), and sorafenib positive control arm (P). **(A,B)**: The expressions of Akt, *p*-Akt (Ser 473), GSK-3β, *p*-GSK-3β (Ser 9), Cyclin D1, and PCNA in mice tumor tissues were examined using western blotting analysis. Representative western blots (**A**) and quantitative analysis of protein expression using ImageJ software (**B**) are shown. **(C,D)**: The expressions of Snail, E-cadherin, N-cadherin, Vimentin, MMP-9 and VEGFA in mice tumor tissues were examined using western blotting analysis. Representative western blots (**C**) and quantitative analysis of protein expression using ImageJ software (**D**) are shown. Protein expression data were normalized to the GAPDH control and are shown as mean ± SD (n = 3). *, *p* < 0.05; **, *p* < 0.01 relative to the X arm (tumor model arm). One-way ANOVA was used to determine statistical significance. **(E)**: IHC showed the inhibition of *p*-Akt (Ser 473), *p*-GSK-3β (Ser 9), Snail, N-cadherin and Cyclin D1, and promotion of E-cadherin levels in BJJP treated mouse tissues, compared with the tumor model arm (X) (scale bar, 100 μm, 200×).

**FIGURE 7:**
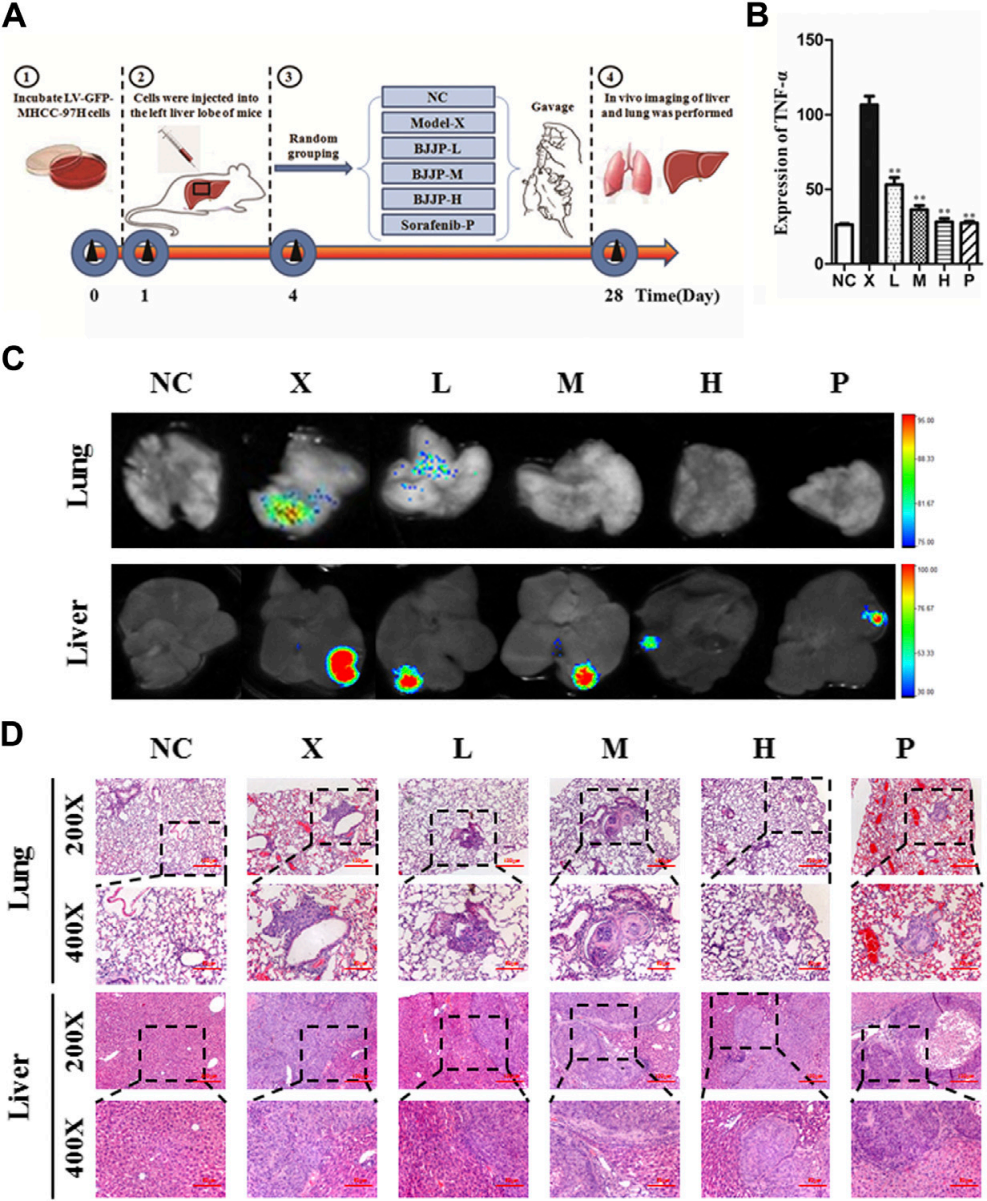
Biejiajian pill (BJJP) decreased lung metastasis of hepatocellular carcinoma *in vivo.* **(A)**: Experimental schematic diagram of liver tumor formation *in situ*. **(B)**: Elisa assay indicated that BJJP significantly decreased the expression of TNF-α in mice serum. **(C)**: Bioluminescence imaging indicated that BJJP notably inhibited lung metastasis of HCC *in vivo.* **(D)**: H&E staining demonstrated that lung metastasis occurred *in vivo*. Mice were randomly grouped into six arms (n = 5): Normal Control arm (NC), Model arm (X), BJJP-low dose arm (L), BJJP-medium dose arm (M), BJJP-high dose arm (H), and sorafenib positive control arm (P). Data are shown as mean ± SD. *, *p* < 0.05; **, *p* < 0.01 relative to the X arm (the tumor model arm). One-way ANOVA was used to determine statistical significance.

To detect the antitumor influence of BJJP *in vivo*, we created a nude mouse model of MHCC-97H xenograft tumor to assess the impact of BJJP in HCC. As shown in Figures 5A–D, the volumes of the tumors were obviously reduced in all BJJP arms (low dose, medium dose and high dose), compared with the model arm (*p* < 0.05). Moreover, all BJJP treatment arms could decrease the expression of KI67 (proliferation marker), compared with the model arm (Figure 5F). In addition, we also investigated the expression of the Akt/GSK-3β/Snail axis and EMT correlated proteins in HCC tumor tissues using western blotting and IHC analyses. As shown in Figures 6A–D, western blotting assays showed that BJJP significantly decreased the N-cadherin, *p*-Akt (Ser473), *p*-GSK-3β (Ser9), Vimentin, CyclinD1, PCNA, MMP-9, VEGFA and Snail expressions, and increased the expression of E-cadherin (*p* < 0.05). Additionally, we inspected the *p*-Akt (Ser473), *p*-GSK-3β(Ser9), Snail, E-cadherin, N-cadherin and Cyclin D1 contents in HCC tumor tissues using IHC. The IHC results were consistent with that of the western blotting analysis (Figure 6E). Based on these results and the inhibitory effects of BJJP on cancer cell invasion and migration *in vitro*, a liver orthotopic transplantation model of HCC was established by injecting GFP-labeled MHCC-97H cells through the left liver lobe of mice. The results showed that the BJJP treated arms remarkably decreased the incidence of lung metastasis, compared with the model arm (Figures 7 C,D). The pulmonary metastasis rate of the low dose, medium dose and high dose BJJP arms were 40, 20 and 20%, respectively, and 80% compared with the tumor in the model arm (*p* < 0.05). The tumor inflammatory microenvironment serves a core function in the progression of hepatocellular carcinoma (Hao et al., 2011). To explore the effect of BJJP on the HCC tumor inflammatory microenvironment, we further analyzed the expression of TNF-α in mice serum using Elisa assay. As shown in Figure 5E and Figure 7B, we found that BJJP could significantly decrease the expression of TNF-α *in vivo* (*p* < 0.05). These data opined that BJJP could repress the carcinogenesis, as well as metastasis of HCC via the Akt/GSK-3β/Snail cascade *in vivo*.

## Discussion

Since the morbidity of HCC has been rising constantly, it is important to inspect potential safe, as well as effective novel treatments for HCC. In China, the primary health care systems, TCM has functioned, and still functions an imperative role because of its unique effects (Hu et al., 2013). BJJP is a well-known composite formula in TCM that can be used to treat HCC, liver cirrhosis and liver fibrosis in clinical practice (Zhang Q. et al., 2019). However, there still is a lack of research on the mechanism of BJJP in the treatment of HCC. Therefore, our research group engaged in research on the mechanism of BJJP in the therapy of HCC. Herein, we first used high resolution mass spectrometry to study the main components of BJJP, and obtained the finger-print of BJJP, which laid a foundation for subsequent research. Moreover, we observed that BJJP suppressed the growth, migration, as well as invasion of HCC *in vitro* and inhibited the growth of subcutaneous tumors and pulmonary metastasis of orthotopic transplantation tumors *in vivo*. Finally, our findings demonstrated that the molecular mechanism, by which the inhibitory effect of BJJP was exerted on the infiltration, as well as metastasis of HCC was related to the inhibition of EMT via the Akt/GSK-3β/Snail cascade.

BJJP is a classical Chinese medicine compound composed of 23 kinds of Traditional Chinese medicine. Due to its complex composition, we used high-resolution mass spectrometry to analyze the main components of BJJP to clarify the mechanism of action of BJJP against HCC. By analyzing the results of mass spectrometry, we obtained 10 main drug components in BJJP, such as Wogonin, Ursolic acid, Zerumbone, 6-Gingerol, Coumarin, Astragalin, Resveratrol, Rutin, Sinapine and Oleanolic acid. Studies have shown that the above 10 pharmaceutical ingredients can regulate the occurrence and development of various tumors via different signaling pathways. Among them, Resveratrol inhibits the invasion and metastasis of colon cancer through reversal of EMT via the AKT/GSK-3β/Snail signaling cascade (Yuan et al., 2019). Moreover, Wogonin (Tian et al., 2014), Ursolic acid (Yan et al., 2010), Zerumbone (Samad et al., 2018), 6-Gingerol (Weng et al., 2010), Coumarin (Zhang E. et al., 2018), Resveratrol (Gao et al., 2020), Rutin (Thomas et al., 2017), Oleanolic acid (Wang et al., 2018) were involved in regulating the proliferation, apoptosis, invasion, as well as metastasis of HCC. Oleanolic acid serves an anti-EMT effect in HCC by increasing iNOS dimerization, NO production, and NT of EMT-related proteins (Wang H. et al., 2019).

Metastasis is the principal cause of cancer-related fatalities, mainly due to limited efficacy of current anticancer treatments for advanced malignancies. Metastatic cascades include the separation of tumor cells from adjacent cells, local infiltration into surrounding tissues (as either singular cells or groups of tumor cells), and tumor entry into nearby pre-existing or newly formed vascular systems. In this case, these aggressive tumor cells are typically typified by partial loss of epithelial biosignatures and the attainment of a mesenchymal-like phenotype, which is usually associated with migratory and invasive property changes that occur during EMT. EMT is one of the key steps in the metastatic cascade, in which cancer cells lose epithelial properties and acquire mesenchymal phenotypes, enabling tumor cells to be aggressive, motile and anti-apoptotic. In addition, activation of the EMT program affects the immunomodulatory properties and immunogenicity of cancer cells, and is associated with immunosuppression induction and acceleration of tumor progression (Lorenzo-Herrero et al., 2018). In recent years, studies have documented that HCC cells going through EMT serve a crucial role in the metastasis of malignant hepatoma cells, thus affecting the recurrence and prognosis of HCC (Giannelli et al., 2016).

EMT serves a pivotal function in the development of metastasis of multiple cancers. Diverse signaling cascades that are atypically activated or inactivated during tumor progression, such as JAK/Stat3, PI3K/Akt, TGF-β/Smad, as well as Wnt/β-catenin, have been found responsible for regulating the EMT process. Previous researches have documented that the Akt/GSK-3β/Snail signaling pathway regulates the invasion and metastasis of various type of tumors, including HCC, via modulating EMT (Cheng et al., 2016). In this pathway, the initiation of Akt represses the activity of GSK-3β, which successively suppresses the Snail phosphorylation, triggering stabilization of the Snail protein, as well as nuclear localization, ultimately promoting EMT. In numerous cells, GSK-3β is inherently active and can dock and phosphorylate Snail to promote its degradation. On the contrary, deactivation of GSK-3β enhances the stabilization of Snail, translocation into the nucleus, as well as ensuing induction of EMT (Qiu et al., 2018). GSK-3β constitutes a multifunctional serine/threonine protein kinase, which serves a pivotal role in the process of EMT. GSK-3β possesses distinct sites of phosphorylation, including Ser9, as well as Tyr216, which play distinct roles in distinct kinds of cancers. Tyr216 phosphorylation activates GSK-3β, whereas Ser9 phosphorylation deactivates GSK-3β. GSK-3β enhances the Snail degradation, whereas *p*-GSK-3β facilitates the Snail nucleation (Qi et al., 2020). Snail, a zinc finger protein, is referred to as an important transcription factor for the regulation of EMT and is involved in the progression, as well as metastasis of several kinds of cancer via regulating EMT. Snail ectopic expression alone can induce EMT and promote the cancer cell motility. Meanwhile, silencing of Snail at least in part represses EMT, then motility is induced by different stimuli. Snail docks to the enhancer of the E-cadherin gene and suppresses its transcription and subsequently downregulates the expression of epithelial biosignatures, E-cadherin, then upregulates the mesenchymal biosignatures, N-cadherin, as well as Vimentin, which are hallmarks of the EMT process (Lan et al., 2018). Moreover, studies have shown that Snail serves an important role in cell cycle and survival. Snail promotes cell survival by suppressing the expression of p53-mediated apoptosis related gene subsets under stress conditions. In addition, Snail also resisted gamma-radiation-induced apoptosis by binding to the PTEN promoter and inhibiting its expression. Knockout of Snail expression in the mouse model of colon cancer could significantly promote cell death (Wu and Zhou, 2010). TAMURA et al. found that E-cadherin could affect proliferation of colorectal cancer stem cells through NANOG (Tamura et al., 2018). In the current study, we observed that BJJP could decrease N-cadherin, *p*-Akt (Ser473), *p*-GSK-3β (Ser9), Snail, as well as Vimentin expressions, while it could increase the expression of E-cadherin, *in vitro*, as well as *in vivo*. The above results revealed that BJJP inhibited HCC proliferation, metastasis and EMT via the Akt/GSK-3β/Snail signaling cascade.

It has been chronicled that Cyclin D1 overexpression is a positive modulator of cell cycle progression that induces cell overgrowth, and is an important marker of malignancy (Li X. et al., 2018). Overexpression of Cyclin D1 causes cells to pass through G1/S checkpoints, thus escalating the percentage of cells in the S phase (Xi et al., 2019). PCNA is a biosignature of cell proliferation that serves a core function in nucleic acid metabolism. Its primary role is in DNA replication, but it also participates in chromatin assembly, DNA excision repair, cell cycle control, as well as RNA transcription (Kang et al., 2019). The cell expression of PCNA remarkably increased in the S, as well as G2 phases of the cell cycle, but was very low in stationary cells, making the protein a good marker for cell proliferation (Shen et al., 2003). Herein, we established that BJJP decreased the expression of Cyclin D1 and PCNA, in MHCC-97H, as well as SMMC-7721 HCC cells. Besides, we inspected the expression of KI67 *in vivo*. KI67 is not expressed in stationary or dormant cells at the G0 stage, but is widely expressed in all proliferating cells (normal and tumor cells), and is also a cell proliferation marker that has been applied as a prognostic factor for the diagnosis of different tumor types (Juríková et al., 2016). Immunohistochemical staining results of the subcutaneous tumor tissues of nude mice showed that BJJP significantly inhibited the expression of KI67, compared with the model arm. The above data implied that BJJP could inhibit the proliferation, as well as growth of HCC by reducing the expression of Cyclin D1, PCNA and KI67.

MMPs are a key factor for the acquisition of infiltration, as well as metastasis characteristics of malignant tumor cells. The migration of tumor cells depends on the secretion, as well as activation of MMPs. Studies have documented that MMPs can serve a pivotal role in the EMT of HCC, and MMP-9 is one of the most inspected MMPs in the pathogenesis of HCC EMT. Overexpression of MMP-9 in HCC can increase lymph node infiltration and promote metastasis, leading to a higher TNM stage, dismal differentiation, and poor overall prognosis (Scheau et al., 2019). MMP-9 serves a critical function in tumor angiogenesis by modulating growth plate angiogenesis and endothelial stem cell recruitment (Bekes et al., 2011). Tumor angiogenesis is the formation of new blood vessels in solid tumors, which provide nutrients and oxygen to promote the proliferation of tumor cells, and is a key step in tumor metastasis. HCC is a highly vascular tumor characterized by neovascularization, and tumor angiogenesis serves a key function in its incidence and development. The expression of vascular endothelial growth factor (VEGF) in human HCC tumor tissues is remarkably greater relative to the vicinal tissues, and is the most important angiogenic factor in HCC (Cheng et al., 2018). The most functional of the VEGF subtypes is VEGFA, which plays an angiogenic role by activating VEGFR2 expression in endothelial cells (Zhang T. et al., 2019). Here, we established that BJJP decreased the expression of MMP-9 and VEGFA, both in MHCC-97H and SMMC-7721 HCC cells.

Growing evidence has shown that chronic and persistent inflammation contributes to cancer development (Hao et al., 2011). HCC is closely linked to chronic hepatitis. TNF-α serves a key function in the activation, as well as amplification of inflammatory responses, which contribute toward tissue destruction, as well as recovery from damage (Hammam et al., 2013). In our assay, we found that BJJP could inhibit serum levels of TNF-α. Moreover, we found that BJJP downregulated the expression of VEGFA, which is a key mediator of angiogenesis in various tumors (Claesson-Welsh and Welsh, 2013). TNF-α has even been reported to mediate macrophage-induced angiogenesis. TNF-α promotes angiogenesis through synergistic VEGF-induced vascular permeability, which is a prerequisite of plasma exudation and the formation of fibrin clots, a matrix that allows angiogenesis. TNF-α also induces gene expression of the proangiogenic molecules of VEGF and its receptors (VEGFRs) (Hammam et al., 2013). Our results revealed that BJJP remarkably reduced the expression of VEGFA and serum TNF-α, which may decrease damage done through tumor inflammation.

In summary, this study uncovered the mechanism by which BJJP inhibited HCC metastasis and invasion. The mechanism involves the modulation of EMT by modulating Snail expression via inhibiting the initiation of the Akt/GSK-3β/Snail signaling cascade. *In vivo*, HCC xenograft data showed that BJJP delayed HCC development and efficiently inhibited lung metastasis. The results of our study indicate that BJJP has the ability to act as an effective treatment agent for HCC.

## Data Availability

The original contributions presented in the study are included in the article/Supplementary Material, further inquiries can be directed to the corresponding authors.
